# Proprioception and Sensorimotor Regulation Across the Day–Night Cycle in Developmental Dyslexia: Toward an Embodied Perspective

**DOI:** 10.3390/brainsci16040346

**Published:** 2026-03-24

**Authors:** Patrick Quercia

**Affiliations:** INSERM UMR1093-CAPS, UFR des Sciences du Sport, Université Bourgogne Franche-Comté, F-21000 Dijon, France; patrick.quercia@u-bourgogne.fr or doc.quercia@gmail.com

**Keywords:** developmental dyslexia, proprioception, sensorimotor regulation, multisensory integration, embodied cognition, sleep–wake cycle, pediatric sleep, neuroplasticity, visuospatial processing, reading acquisition

## Abstract

**Highlights:**

**What are the main findings?**

- Children with developmental dyslexia may exhibit alterations in proprioceptive processing associated with differences in postural control, visuospatial localization, and multisensory tasks.
- Experimental observations suggest that sensorimotor regulation across the sleep–wake cycle may influence multisensory stability and conditions relevant to reading acquisition.

**What are the implications of the main findings?**

- Proprioception may represent a complementary dimension contributing to the heterogeneity of dyslexic profiles alongside established phonological and cognitive models.
- Integrating sensorimotor regulation and sleep–wake dynamics within an embodied framework may open new directions for research and multidisciplinary approaches to reading difficulties.

**Abstract:**

**Background**: Sensorimotor differences have frequently been reported in children with developmental dyslexia, but are often considered secondary or comorbid to phonological deficits. Within an embodied cognition perspective, reading acquisition emerges from dynamic interactions between bodily regulation, multisensory integration, and learning-related neural plasticity. Proprioception contributes to spatial orientation, motor coordination, and perceptual stabilization, while sleep-dependent processes play a critical role in the consolidation and automatization of cognitive and motor skills. **Objectives**: Building on early clinical observations, including the hypothesis proposed by Martins da Cunha, this review explores whether variations in proprioceptive processing and sensorimotor regulation may influence multisensory stability and the conditions under which reading skills develop in some individuals with dyslexia. **Methods**: This narrative synthesis integrates clinical observations and experimental paradigms examining proprioceptive function in children with dyslexia, including studies conducted in our laboratory over the past two decades. These investigations address postural regulation under varying attentional demands, laboratory measures of proprioceptive acuity, visuospatial localization tasks, multisensory interactions, and exploratory observations concerning sleep–wake regulation. **Results**: Across studies, children with dyslexia often show differences in proprioceptive processing associated with variations in postural regulation, visuospatial stability, and multisensory tasks. Laboratory measurements suggest reduced proprioceptive acuity in some individuals, with moderate correlations observed between proprioceptive sensitivity and reading-related measures. Additional observations suggest that nocturnal physiological regulation—including respiratory dynamics and sleep architecture—may interact with daytime sensorimotor stability and attentional functioning. **Conclusions**: Taken together, these findings support the hypothesis that variations in sensorimotor regulation across the sleep–wake cycle may influence the stability of multisensory processing and attentional conditions relevant for reading acquisition. Within this perspective, proprioception is not proposed as an alternative explanation for dyslexia but as a complementary dimension that may contribute to the heterogeneity of dyslexic profiles. Further longitudinal and controlled studies are required to clarify the relationships between sensorimotor regulation, sleep-dependent plasticity, and learning processes.

## 1. Introduction

Developmental dyslexia is classically defined as a neurodevelopmental disorder with a strong genetic component, most commonly associated with a phonological processing deficit [1]. Alternative hypotheses include the cerebellar theory, emphasizing impaired automatization of motor and cognitive skills; the visuo-attentional hypothesis, which highlights differences in attentional allocation and visual processing; and the magnocellular theory, attributing reading difficulties to alterations in visual motion processing [2,3,4].

These theoretical perspectives have provided important insights into the cognitive and neural mechanisms underlying reading acquisition. However, the considerable heterogeneity observed across dyslexic profiles suggests that multiple interacting factors may contribute to reading difficulties.

Developmental dyslexia frequently co-occurs with other neurodevelopmental conditions, including attention-deficit/hyperactivity disorder and developmental coordination disorder. Epidemiological studies indicate that such comorbidities are common and may contribute to the heterogeneity of dyslexic profiles [1]. Within this broader neurodevelopmental landscape, sensorimotor differences observed in some individuals with dyslexia may partly overlap with mechanisms also implicated in these associated conditions. Although sensorimotor processes have occasionally been considered within existing theoretical frameworks—particularly in cerebellar accounts—their potential contribution to the broader regulation of multisensory integration and bodily control remains comparatively underexplored.

The objective of this article is not to propose a new single-deficit model of developmental dyslexia, but to explore the hypothesis that variations in sensorimotor regulation—particularly proprioceptive processing—may influence multisensory stability and the conditions under which reading is acquired. To examine this possibility, the manuscript first reviews conceptual foundations linking proprioception and sensorimotor regulation, then summarizes experimental and clinical evidence related to postural control, proprioceptive acuity, and multisensory integration in dyslexia, before extending the discussion toward sleep–wake regulation and finally proposing an integrative embodied perspective on dyslexia.

This integrative perspective is guided by several principles that frame the present synthesis.

This approach is guided by five core principles:

1. A necessary return to clinical foundations. Careful attention to bodily clinical signs and comprehensive observation of the dyslexic child precede and guide fundamental research. Progress requires the coordinated use of complementary skills, combining clinical expertise with laboratory-based investigation.
2. Integration of existing theories. No theoretical account of dyslexia—whether sensory, motor, or cognitive—is excluded or privileged, as each is supported by substantial empirical evidence.
3. Embodied cognition. Brain functions are examined through their continuous sensorimotor interactions with the body and the environment, consistent with contemporary embodied cognition frameworks in cognitive science and neuroscience [5]. Within this perspective, cognitive processes emerge from dynamic interactions between neural activity, bodily states, and environmental constraints, rather than from isolated cerebral mechanisms. Proprioception, therefore, plays a central integrative role, not merely as a passive sensory modality but as a key contributor to the regulation of perception, action, and learning. This view has important implications for understanding language acquisition, creative expression, and multimodal learning.
4. Rethinking comorbidities. Rather than being conceptualized as independent disorders, comorbidities may represent different manifestations of shared neurodevelopmental mechanisms, suggesting possible common underlying factors that warrant investigation.
5. Temporal dynamics of neurodevelopment. Neurogenesis and brain plasticity follow specific temporal dynamics that require consideration of both ontogenetic stages and nychthemeral phases, given the critical role of sleep in memory consolidation and cognitive automatization [6].

These principles provide the conceptual framework for the following sections, which review empirical findings related to sensorimotor regulation in dyslexia before proposing a broader integrative interpretation.

## 2. Conceptual Background: Proprioception and Sensorimotor Regulation

Early clinical observations suggested that disturbances in postural regulation and sensorimotor integration might accompany certain reading difficulties [7,8]. In the 1970s, Kohen-Raz reported postural instability in children with reading difficulties using electronic ataxiametry [9], and Martins da Cunha described Postural Deficiency Syndrome, characterized by stereotyped postural asymmetries associated with altered proprioceptive and visual integration [10]. To emphasize that the manifestations of proprioceptive dysfunction extend beyond postural control, Postural Deficiency Syndrome has been redefined within the framework of our clinical investigations as Proprioceptive Dysfunction Syndrome, while preserving the acronym PDS.

Proprioception relies on a hierarchically organized system of mechanoreceptors in muscles, tendons, and joint capsules—including muscle spindles, Golgi tendon organs, and Ruffini and Pacini corpuscles [11]—as well as afferent pathways that encode body position and movement. These receptors are particularly dense in regions requiring fine motor precision, such as the fingers, cervical muscles, and extraocular muscles [12].

Recent advances in sensory neurobiology have highlighted the central role of the PIEZO2 mechanotransduction channel in proprioceptive signaling [13,14]. Experimental studies in animal models have further shown that disruptions in proprioceptive pathways, including PIEZO2 and ASIC2 signaling, can affect spinal alignment and postural organization in mice [15,16].

Proprioception provides continuous information on limb position, muscle tension, and joint orientation, but also contributes to directional encoding of exteroceptive modalities, particularly vision and audition [17]. By linking directional information from spatially distant receptors, proprioception ensures coherent sensorimotor interaction with the environment [11]. Gamma motor neurons dynamically regulate muscle spindle sensitivity, indicating that proprioception is actively modulated [12]. During movement, the CNS compares incoming proprioceptive feedback with predicted sensory consequences from an efference copy, generating corrective responses and supporting motor and sensory learning and automatization [11].

Since 2002, our laboratory has investigated relationships between proprioception, cognition, and dyslexia. Early observations revealed substantial heterogeneity in the literature regarding motor difficulties in children with dyslexia, likely due to disciplinary fragmentation between clinical and psychological studies employing distinct methodologies [8]. We adopted an integrative approach considering perception, action, and cognition as inseparable processes, hypothesizing that proprioceptive dysfunctions may disrupt these interactions and contribute to developmental cognitive disorders such as dyslexia.

This article presents a narrative synthesis integrating experimental paradigms, previously published studies from our research program, and clinical observations collected over several decades. Its aim is to explore the hypothesis that variations in proprioceptive regulation may influence multisensory stability and the learning processes involved in reading acquisition in developmental dyslexia. Because the literature addressing proprioception in dyslexia remains fragmented across several disciplines, a narrative synthesis was considered more appropriate than a formal systematic review.

## 3. Empirical Evidence on Sensorimotor Regulation in Dyslexia

Within embodied cognition frameworks, proprioception plays a central role in establishing the sensorimotor reference frames that allow the integration of visual, vestibular, and somatosensory signals [5]. From this perspective, alterations in proprioceptive processing may influence the stability of multisensory integration, which is essential for spatial perception and for visually guided behaviors such as reading.

The following sections summarize experimental paradigms and empirical observations that explore the potential role of proprioceptive regulation in postural control, motor imagery, and multisensory integration in dyslexia.

### 3.1. Postural Control Deficits in Dyslexia: A Review of the Literature

Postural regulation and balance maintenance are established models of sensorimotor automatization, relying on proprioception to integrate vestibular, ocular, oral, and plantar inputs [8].

Studies investigating postural control in dyslexia frequently report differences in balance regulation compared with typically developing readers. Some experimental studies have described relatively high rates of postural instability in a large proportion of dyslexic populations [18].

However, the literature remains heterogeneous. Several meta-analyses and systematic reviews have highlighted substantial variability across studies, likely reflecting methodological differences, sample characteristics, and the heterogeneity of dyslexic profiles [7]. In some investigations, the relationship between postural control and reading performance appears weak or indirect, suggesting that postural differences may characterize specific subgroups rather than representing a universal feature of dyslexia [8].

Consequently, postural instability should not be interpreted as a defining marker of dyslexia but rather as a sensorimotor characteristic that may be present in some individuals and that may interact with other cognitive and perceptual factors involved in reading acquisition.

### 3.2. Evidence Investigating the Postural-Dyslexia Nexus: Clinical Evidence

The observations described in this section originate primarily from clinical examination and long-term clinical practice. While they have motivated several experimental paradigms, they should be interpreted as descriptive clinical observations rather than as experimentally established mechanisms.

#### 3.2.1. Phenotypical Markers of Postural Disorders in Dyslexic Children

**Figure 1:**
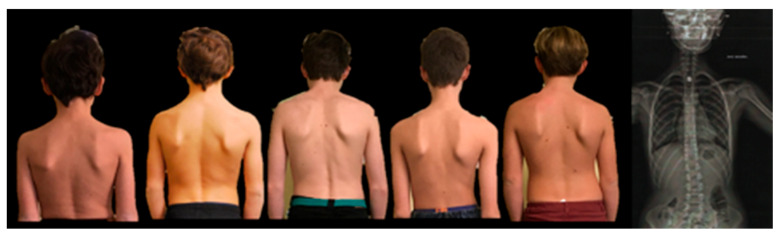
Children with dyslexia consistently exhibit a scoliotic postural attitude, associated with asymmetrical paravertebral muscle tone, suggesting altered sensorimotor integration and postural control mechanisms.

PDS was initially characterized through clinical observation and patient-reported muscular, spatial, and cognitive signs [10]. To further investigate these manifestations, we administered Martins da Cunha’s questionnaire to 60 dyslexic children of both sexes, all presenting with characteristic reading delays [19]. Although responses were heterogeneous, clinical examination consistently revealed a set of physical signs across all participants, including scoliotic posture, asymmetric muscle tone, reduced unilateral head rotation, ocular convergence asymmetry, and plantar support imbalance (Figure 1). Notably, visual-perceptual disturbances emerged under conditions of binocular disruption, accompanied by altered body schema perception and impaired eye–hand coordination.

Photographic analysis did not reveal a single postural archetype; rather, the only consistent finding was maximal heel loading, measurable via calibrated plantar pressure sensors. This suggests that children perceived upright posture predominantly when pressing on their heels, which in turn created a tendency toward backward instability.

Such perceptual errors are consistent with proprioceptive dysfunction. Experimental studies have shown that targeted disruptions of proprioceptive input—via vibrations applied to postural muscle chains, cervical, oculomotor, and masticatory muscles, as well as the plantar surface—can induce illusions of body displacement and alter the subjective postural vertical [20,21,22,23,24]. The subjective postural vertical reflects the perception of body orientation relative to gravity. While highly sensitive to variations in proprioceptive input, it also relies on multisensory integration of proprioceptive, vestibular, and gravitational cues, with a supplementary contribution from vision. When this integrative process is disrupted, a mismatch arises between the actual posture and the posture perceived by the individual. Under gravitational forces, the child is thus objectively in a state of posterior instability—akin to an impending backward fall—that is not consciously perceived but continuously compensated for by the nervous system. The observed postures likely represent compensatory strategies distributed across multiple levels and spatial planes, including anterior pelvic tilt with hyperlordosis and forward head posture with kyphosis.

These postural adjustments appear to follow a pattern that may be described as a “compensation conservation principle.” In clinical observation, pronounced compensation at one anatomical level often appears to reduce the need for compensatory adjustments elsewhere (for example, marked forward head projection may lessen anterior pelvic tilt, and vice versa).

It should be emphasized that this notion currently represents a heuristic clinical model derived from repeated observations, rather than a quantitatively validated biomechanical law. Future studies using quantitative posturographic and biomechanical analyses would be required to formally test this hypothesis.

**Figure 2:**
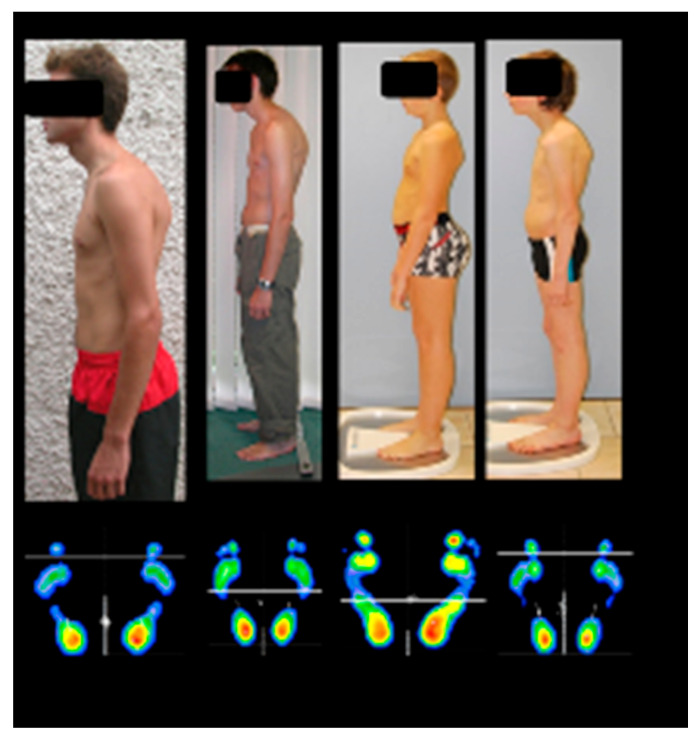
To compensate for a perceived risk of backward instability, potentially related to inaccurate proprioceptive information, individuals adopt postural strategies such as anterior pelvic translation, forward head displacement, or a combination of both. Podometric measurements show a predominance of plantar support in the posterior regions, particularly at the heels.

Compensation may be distributed between the upper and lower body, resulting in relatively balanced postures, but abnormally high heel pressure persists (Figure 2). The heterogeneity of compensatory patterns has three major implications. First, postural anomalies may be subtle to untrained observers, especially when foot support is not assessed. Second, this variability may explain the low reliability of simple exploratory maneuvers, such as the pull test used in cerebellar deficit studies [25]. Third, the diaphragm, beyond its respiratory role, functions as a postural stabilizer; individual differences in diaphragmatic engagement affect trunk control, postural stability, and motor coordination [26,27]. This concept is essential for understanding the link between learning difficulties and proprioceptive dysregulation.

#### 3.2.2. Stabilometric Analysis: Force Platform Assessments of Balance

Building on these clinical observations, we assessed postural stability in 50 dyslexic children and 38 typically developing readers using a force platform [28]. The force platform records ground reaction forces and computes the center of pressure (CoP), from which anteroposterior and mediolateral displacements, as well as sway amplitude, velocity, and area, are analyzed. It is widely used in postural, locomotor, and balance research [29].

All dyslexic children exhibited at least one abnormal postural parameter, particularly along the anteroposterior axis. They also struggled with single-leg stance, especially with eyes closed, highlighting a strong reliance on visual input for postural control.

#### 3.2.3. Dyslexia Dual-Task Interference: The Impact of Cognitive Load on Stability

We next examined the influence of cognitive load on postural stability. Three groups were compared: 12 untreated dyslexic children, 15 dyslexic children who received a proprioceptive intervention (mean duration: 14.5 ± 11.8 months), and 12 typically reading controls [30]. During the control condition, children fixated on a single point on an A4 sheet 40 cm away. In the experimental condition, the point was replaced by a 50-cell matrix with color words printed in incongruent ink colors (four colors, randomized), creating a pseudo-Stroop task to increase attentional and inhibitory demands while standardizing oculomotor strategies. Children freely searched for two target words without time constraints to minimize stress effects. Analyses focused on CoP velocity and confidence ellipse area, key indicators of postural adjustment.

Dyslexic children showed significantly higher CoP velocity during the pseudo-Stroop task. ANCOVA confirmed this effect was associated with group membership rather than age (*p* = 0.008), with post hoc analyses indicating that only dyslexic children exhibited this increase (*p* < 0.01). CoP sway area was also larger in dyslexic children (*p* = 0.02).

In this exploratory context, dyslexic children who had previously received a proprioceptive intervention showed lower CoP velocity and sway area than untreated dyslexic children, reaching levels comparable to those of typically reading controls.

This effect persisted even when testing occurred one hour after discontinuing the intervention, suggesting durable recalibration of postural control.

However, treatment duration varied widely among participants (mean duration: 14.5 ± 11.8 months), reflecting the clinical and individualized nature of the intervention. Because the study did not include randomization, placebo control, or maturation controls, these observations should not be interpreted as evidence of a causal treatment effect. Rather, they suggest that sensorimotor training may influence postural regulation in some individuals, a hypothesis that would require confirmation through controlled longitudinal studies.

#### 3.2.4. Sensory Modulation: Effects of Proprioceptive Manipulation on Balance

Postural control provides an indirect measure of proprioceptive activity, but proprioception can also be probed directly using tendon vibration. This technique selectively activates muscle spindles, induces movement illusions, and perturbs balance [31], thereby allowing a more precise assessment of the contribution of proprioception to postural regulation.

In the present study, ankle co-vibration was applied while children stood on a force platform in order to examine interactions between proprioceptive perturbation and cognitive–attentional load [32]. Two experimental conditions were tested: a control condition, in which children fixated a point located 40 cm in front of them, and an attention condition in which they visually scanned a sheet containing stars of two different sizes while keeping their head still and silently counting the stars of a target size. No time constraints were imposed.

Three groups were compared: 30 untreated dyslexic children, 42 typically developing controls, and 51 dyslexic children who had previously received proprioceptive training. The duration of this clinical intervention varied substantially between participants (mean duration: 16.9 ± 17.9 months), reflecting the individualized nature of the clinical management. Consequently, the present findings should be interpreted as exploratory observations rather than as controlled evidence of treatment efficacy.

Ankle co-vibration produced a greater destabilizing effect on balance in dyslexic children than in controls, whether treated or untreated, suggesting differences in proprioceptive integration. Although the training did not completely eliminate this difference, treated children showed improved performance under attentional demand, which may indicate a reduced need for compensatory effort and a relative release of cognitive resources.

### 3.3. Quantifying Proprioceptive Acuity in Dyslexic Children: Laboratory Metrics

**Figure 3:**
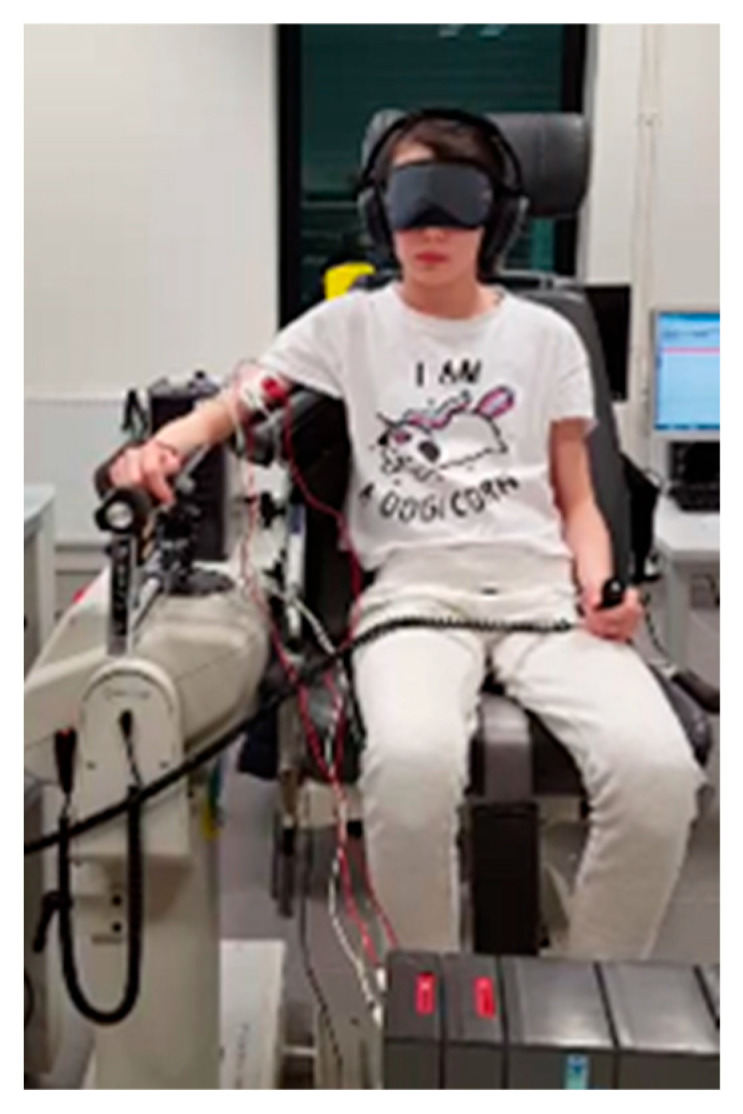
Arm proprioceptive acuity was assessed through repeated measurements using a computerized ergometer under laboratory conditions. Auditory and visual inputs were eliminated, and the absence of voluntary arm activity was verified using surface electromyography. Participants were instructed to indicate the perception of passively induced arm movement by pressing a response button with the contralateral hand.

While postural measures provide indirect insight into proprioceptive function, accurate assessment requires tools independent of postural reactions or efference copy. To this end, we employed a laboratory digital ergometer to record the time needed to detect passive limb movements, with EMG monitoring ensuring no active muscle involvement [33]. This device, although precise, is costly and impractical for routine clinical use (Figure 3).

In this experimental paradigm, children with dyslexia exhibited reduced proprioceptive acuity compared with typically developing readers, with longer and more variable detection times during slow passive elbow movements.

A proprioceptive acuity index combining normalized reaction time and variability showed a moderate but statistically significant correlation with reading and phonological measures (r = 0.45, *p* = 0.008), indicating an effect of modest magnitude in this sample. These findings suggest a possible relationship between proprioceptive sensitivity and reading-related abilities in this sample.

However, the sample size remained relatively small, and the magnitude of the correlations should therefore be interpreted cautiously. In addition, these results originate from a limited number of experimental studies and have not yet been independently replicated in other laboratories.

Consequently, while these findings provide preliminary support for the hypothesis of altered proprioceptive processing in some individuals with dyslexia, larger-scale studies including standardized effect-size reporting and independent replication will be necessary to confirm their robustness.

Additional analyses were conducted to examine the robustness of these correlations. The indices did not deviate significantly from normality when pooled (*p* > 0.2), suggesting that the observed correlations were unlikely to be driven by a bimodal distribution. Separate analyses revealed a moderate correlation in dyslexic children (R = 0.52, *p* = 0.03), but not in controls (R = 0.15, *p* = 0.57). These results suggest that variations in proprioceptive processing may be associated with reading performance in the dyslexic group.

In a subgroup of 12 participants (6 dyslexics, 6 controls), proprioceptive acuity measured at both the elbow and hip correlated with reading ability in dyslexic children (Spearman R = 0.83, *p* = 0.04) but not in controls (R = 0.03, *p* = 0.96). Although based on a very small sample, these findings suggest that the observed proprioceptive differences may reflect a broader sensorimotor characteristic rather than an effect directly related to reading practice, as neither the elbow nor the hip is directly involved in reading tasks.

### 3.4. Mental Representations of Action: Motor Imagery in Dyslexia

Motor imagery (MI) is the mental simulation of an action without overt execution [34]. MI actively recruits neural representations overlapping those engaged during actual movement, preserving temporal and spatial features such as trajectory, amplitude, and timing [35]. This indicates that the brain simulates movement dynamics even without peripheral output.

MI also engages sensory components, particularly kinesthetic and proprioceptive modalities, allowing the individual to mentally experience movements as if performed [36]. Functional neuroimaging confirms activation of cortical and subcortical motor areas during MI [37]. Importantly, MI follows the principle of isochrony (Fitts’ law): imagined movement durations closely match actual execution, reflecting the fidelity of internal motor representations [38,39].

We applied MI paradigms to 18 adolescents with developmental dyslexia (without dyspraxia or ADHD) and 18 age-matched controls [40]. Participants performed a visually guided pointing task, then imagined performing it, indicating estimated movement durations. Mental representation was assessed by (1) compliance with Fitts’ law, predicting longer movement times for smaller targets, and (2) preservation of isochrony between executed and imagined actions.

Compared with controls, dyslexic adolescents exhibited significantly longer movement times in both executed and imagined conditions. All dyslexic participants showed altered mental representations, with deviations from Fitts’ law predictions and disrupted isochrony. Across groups, motor imagery performance correlated significantly with combined reading scores for words and pseudowords, including subtests specifically assessing phonological processing.

These findings are consistent with the hypothesis that altered sensorimotor representations may contribute to differences in motor imagery performance. Because motor imagery engages internal models of movement that integrate proprioceptive and kinesthetic information, these results may reflect differences in proprioceptive processing affecting the internal simulation of action.

However, alternative interpretations should also be considered. In particular, cerebellar theories of dyslexia emphasize differences in motor timing, prediction, and automatization, functions that are also critically involved in motor imagery tasks [2,37]. Consequently, the present findings cannot distinguish between proprioceptive and cerebellar explanations and should be interpreted as evidence of altered sensorimotor representations rather than as specific support for a single mechanism.

### 3.5. Vertical Maddox Test as a Proprioceptive Stress Test of Binocular Alignment and Visuospatial Localization

Efficient reading relies on accurate visuospatial localization, which ensures stable binocular fixation and appropriate coordination of saccadic eye movements [41]. Although spatial localization emerges from complex sensorimotor learning, binocular fixation is inherently imperfect, with frequent horizontal and vertical disparities, particularly in children. Dyslexic children typically exhibit longer and less stable fixations, shorter progressive saccades, and more frequent regressive saccades, reflecting an oculomotor pattern similar to that of younger typically developing readers and suggesting delayed central maturation. Word identification requires precise foveal projection (1–2° of visual angle), while parafoveal vision (up to ~5°) provides essential spatial and prelexical information for saccade planning [42].

Because binocular alignment depends on the integration of visual, vestibular, and proprioceptive signals, subtle vertical misalignments may provide a useful window into the stability of sensorimotor integration. In this context, the Vertical Maddox Test (VMT) was used not as a diagnostic marker of dyslexia, but as an exploratory paradigm to investigate the dynamic regulation of binocular alignment under multisensory perturbations [43]. In particular, the test can be viewed as a proprioceptive stress test, as it destabilizes binocular fusion while minimizing the contribution of voluntary oculomotor commands.

Visuospatial localization results from the combined contributions of foveal binocular fusion, motor efference copy, and ocular proprioceptive feedback [44]. To specifically assess the potential contribution of proprioceptive dysfunction, it is therefore necessary to dissociate this component from the other two. A simple tool used in clinical ophthalmology for more than a century is the Vertical Maddox Test [45], which allows the detection of subtle vertical misalignments between the two eyes. Small vertical deviations can also be observed in the general population, and their functional significance remains debated.

**Figure 4:**
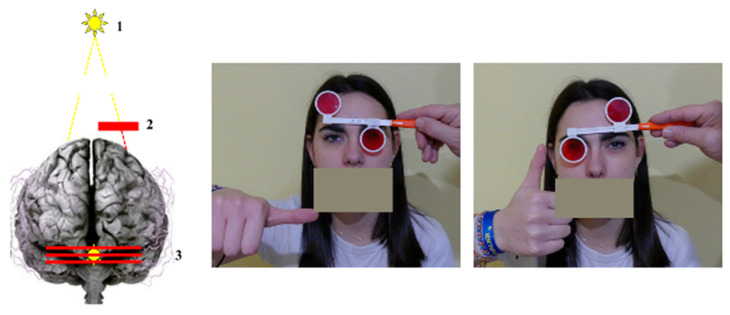
VMT Principle. The child fixates on a light point placed 3 m away (1). One eye looks directly at the point, while the other sees it through a red screen (2) made of very strong vertical prisms. This screen turns the light point into a horizontal red line. The brain then fuses the two images, so the child perceives a red line crossing the light (3). When the point’s size and brightness are properly controlled, the perceptible offset between the light edge and the red line is about 0.75 diopters (0.37°). To avoid altering signals carried by the trigeminal nerve—which transmits both ocular proprioceptive information and sensory input from the oral region—the child indicates the line’s position with their thumb, without speaking.

The test involves placing a red screen composed of high-power vertical prisms in front of one eye while the subject binocularly fixates a point light source located approximately 3 m away at eye level (Figure 4). The screen transforms the point light source into a horizontal red line. Provided that the light source is precisely calibrated (0.7 mm diameter, 100 lumens at a distance of 2 cm), the resulting line is sufficiently thin to allow the subject to report with precision (0.75 prism diopters—approximately 0.37°) whether it is perfectly aligned with the white light perceived by the other eye, or slightly displaced upward or downward. Such misalignments are termed vertical heterophoria.

The VMT therefore disrupts binocular fusion by presenting different images of the same object to each eye. This perturbation is particularly pronounced because the point light is primarily perceived by the macular region of one eye, whereas the red line is processed by the macula as well as by a large portion of the peripheral retina of the other eye. The test also minimizes the influence of efference copy signals, which are engaged only when a potential motor command is generated. Importantly, the oculomotor system lacks independent vertical motor commands for a single eye that could correct vertical misalignment between the two eyes [45]. These conditions create an optimal context for assessing the contribution of ocular proprioception to binocular stability and for examining whether distal proprioceptive inputs can modulate its function.

The relevance of this paradigm has been validated in studies investigating the relationship between vision and postural control [46]. It offers several methodological advantages: it is easy to administer, rapid, inexpensive, widely accessible in clinical practice, and provides meaningful information when implemented using a rigorous protocol.

In the present work, the VMT was used as an experimental paradigm to explore the stability of visuospatial localization under multisensory perturbations rather than as a diagnostic marker of dyslexia. Our objective was to examine whether subtle variations in binocular alignment might reveal differences in sensorimotor integration when distal sensory inputs are manipulated.

#### 3.5.1. Disrupted Visuo-Spatial Localization in Dyslexic Children

**Figure 5:**
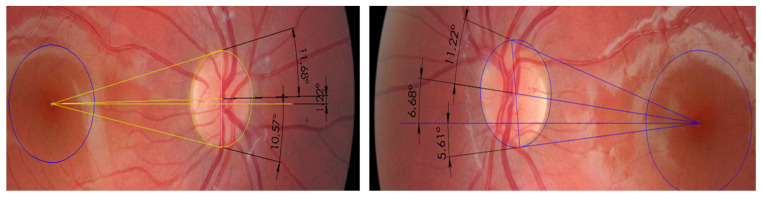
Ocular torsion measured by fundus photography, showing extorsion of the left eye compared with the right eye.

We assessed visuo-spatial localization in 42 children with dyslexia using the VMT, comparing them to 22 typically developing controls [43]. All participants had normal ophthalmological examinations and were medication-free. Neuropsychological profiling identified 14 children with surface dyslexia, 4 with pure phonological dyslexia, and 24 with mixed dyslexia. The VMT was administered monocularly for each eye, with binocular recovery between tests, across nine conditions designed to probe ocular proprioception and its stability during distal sensory stimulation: natural sitting posture without foot support; head tilt to each shoulder (Bielchowsky maneuver) engaging oblique muscles; upright sitting altering spinal proprioception; tongue or lip manipulations targeting trigeminal or facial inputs; standing posture with and without foam insole to modulate plantar feedback. When vertical deviations were detected, low-power prisms were used for correction, either vertical or along oblique muscle axes. Subjective torsion was measured with the double Maddox rod test, and fundus photography assessed objective foveal torsion relative to the optic disc center (Figure 5).

Children with dyslexia in our cohort frequently exhibited small vertical heterophorias detectable with the VMT. Such micro-deviations can also be observed in typically developing individuals. However, in our cohort, the vertical heterophorias observed in dyslexic children displayed features that differed markedly from those observed in control children (*p* < 0.0001):

- **Constancy:** the deviation was consistently present across dyslexia subtypes.
- **Low magnitude:** typically ranging from 0.25 to 0.75 prism diopters, below the detection threshold of the standard cover test but above the physiological range of 0.10–0.16° described by Van Rijn [47]. Compensation through voluntary control or the use of low-power vertical prisms proved ineffective.
- **Lability:** the vertical deviation was highly labile across the nine testing conditions, shifting in response to sensory and postural cues, particularly trigeminal and plantar inputs [48,49]. A lability index can be calculated by assigning one point for each change in spatial position associated with a given sensory stimulation.
- **Oblique muscle imbalance:** the deviations appeared consistent with a tonic imbalance of the oblique muscles, accompanied by both subjective and objective cyclotorsion. Bielschowsky maneuvers frequently revealed inferior oblique hypertonicity or occasional superior oblique restriction, consistent with minimal Brown-like patterns [45]. Vertical misalignment corrections, which cannot be voluntarily produced in a controlled manner, likely reflect subtle changes in oblique muscle tone. These small eye movements appear to be associated with postural reflex mechanisms linked to the perception of the subjective vertical rather than voluntary control [50].
- **Efficacy of bilateral oblique prisms:** when prescribed based on combined ophthalmological and postural assessments, these prisms eliminated vertical heterophoria, lability, and cyclotorsion in most cases. Effective correction often requires asymmetric muscle relaxation along axes differing from anatomical norms, highlighting the contribution of orbital mechanics.

To our knowledge, this specific combination of features has not been clearly described in previous studies. However, these characteristics should not be interpreted as a specific marker of dyslexia. Rather, within the present paradigm, they provide a means of exploring the dynamic stability of binocular alignment under multisensory perturbations, potentially offering indirect insights into sensorimotor integration processes.

Complementary eye-tracking studies based on iris-movement recordings showed that in 58% of trials the eyes remained stationary while participants reported displacement of the red line, consistent with the proprioceptive models proposed [51,52]. According to these models, distal vibrations do not move the eyes but instead modulate afferent signals, generating a kinesthetic illusion. The protocol has therefore been termed the “Perceptual Maddox Test,” emphasizing sensory modulation rather than mechanical eye movements.

Before proprioceptive treatment, heterophoria lability did not correlate with reading performance, indicating the test primarily reflects proprioceptive integrity. It becomes clinically valuable for monitoring interventions.

**Figure 6:**
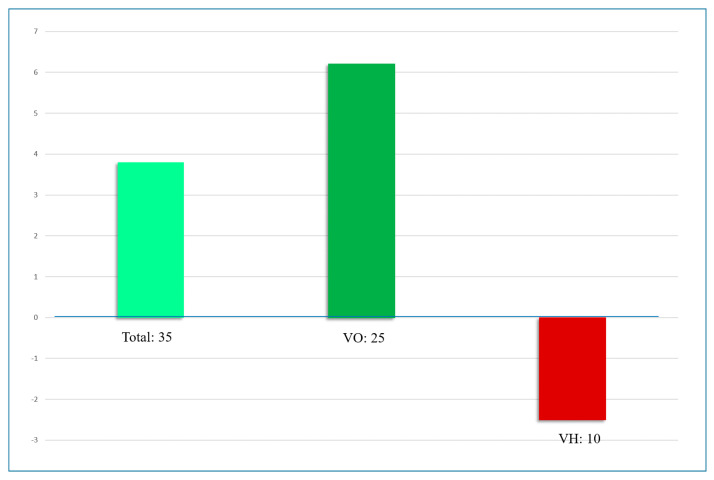
Evolution of reading level (expressed in months) following a 3-month proprioceptive treatment, categorized by VMT. Dyslexic children with vertical orthophoria (VO) progressed significantly more than those remaining in vertical heterophoria (VH). (*p* = 0.005): +6.48 months vs. –2.8 months.

In a cohort of 35 dyslexic children undergoing proprioceptive treatment, reading performance improved on average by 3.4 months over 3.6 ± 0.6 months, with high inter-individual variability [53]. Children who achieved vertical orthophoria without detectable lability during sensory perturbations showed greater improvements in reading level than those with persistent vertical heterophoria (Figure 6).

However, these observations were obtained in a non-randomized clinical context, without a placebo control or comparison of developmental trajectories. Consequently, the relationship between ocular alignment changes and reading improvement cannot be interpreted as evidence of a causal therapeutic effect. Rather, these findings suggest a possible association that would require confirmation in controlled longitudinal studies.

#### 3.5.2. Disrupted Multisensory Integration Under Experimental Proprioceptive Modulation in Developmental Dyslexia

Learning to read requires the integration of auditory and visual information. In children with dyslexia, in whom proprioceptive status has generally not been considered, multisensory deficits appear to arise primarily from unisensory auditory and visual impairments rather than from deficits in multisensory integration per se. These deficits are associated with temporal processing abilities and reading skills [54].

Difficulties in simultaneous auditory processing, combined with a reduced visual attention span, further suggest the involvement of an amodal cognitive mechanism underlying certain dyslexia profiles. This hypothesis is supported by longitudinal data indicating that approximately 30% of children with dyslexia exhibit sensory deficits concomitant with impairments in cognitive skills essential for reading [55,56].

**Figure 7:**
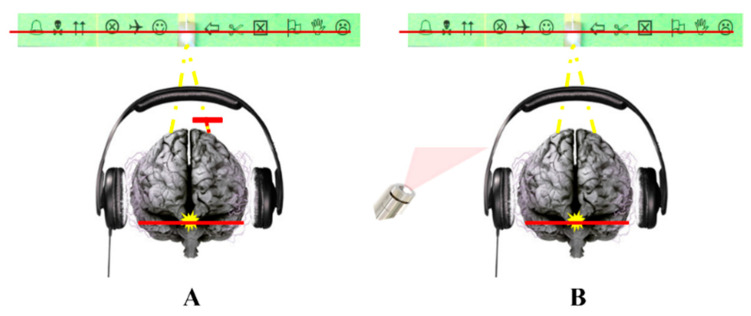
Visual pseudo-scotomas were assessed using a vertical Maddox rod test performed monocularly, with the device placed in front of each eye separately (**A**), or under binocular viewing conditions using a red laser beam (**B**). In both paradigms, cortical visual input is equivalent, consisting of a red vertical line aligned with a point light source. Illustrations positioned on either side of the light indicate the perceived areas of visual field loss.

To examine whether proprioception, as a directional link across sensory modalities, contributes to these deficits, we conducted two experimental studies comparing children with developmental dyslexia to typically developing reader children [57,58]. Fourteen untreated children with dyslexia and ten age-matched controls, all carefully assessed for binocular vision and ophthalmological health, completed multisensory integration tasks combining either visual and auditory information or simultaneous visual information and proprioceptive modifications at a distance. Binocular vision was manipulated using the VMT, as described previously. To ensure that the induced visual disturbance was specifically related to a modification of ocular proprioception via the VMT, a control condition reproducing the same visual percept while preserving retinal fusion was introduced (a horizontal red line generated by a red laser crossing the light source, without a Maddox screen placed in front of either eye). This paradigm allowed comparison between two perceptually equivalent situations differing only in the state of ocular proprioception (Figure 7).

In the conditions exploring audiovisual interactions, participants were exposed to binaural auditory stimuli (500 or 1000 Hz, 50 dB, 500 ms) while observing the visual stimulus consisting of a red line intersecting a light source. Participants were instructed to report whether the visual image remained intact or whether visual losses occurred during auditory stimulation, and to indicate their spatial location when present. These transient visual losses were termed visual pseudoscotomas (VPS) to emphasize their functional and reversible nature, in contrast to visual scotomas resulting from organic lesions of the visual pathways. A total of 45 trials were administered, with standardized rest periods between stimulations.

**Figure 8:**
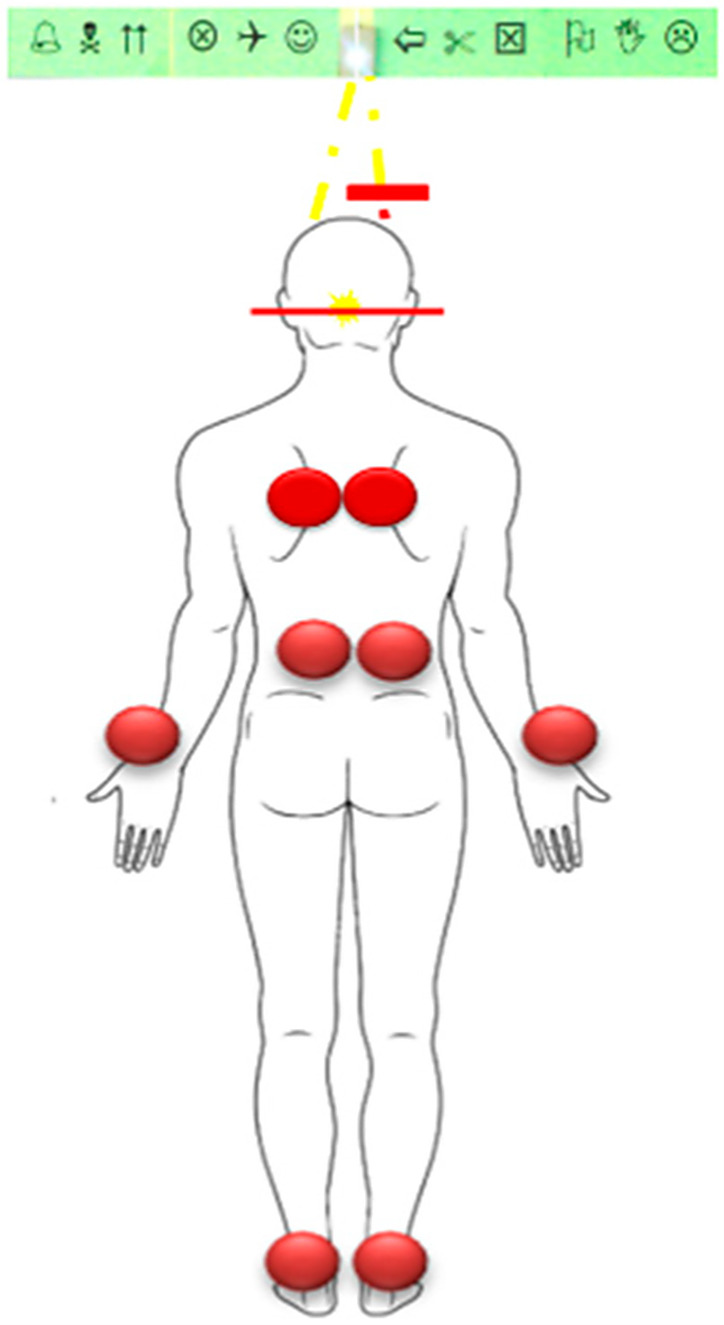
Direct proprioceptive stimulation was delivered to muscles involved in postural control (paravertebral and ankle muscles) as well as to muscles not primarily engaged in postural regulation (wrist muscles).

In the visuoproprioceptive task, which explored interactions between vision and general proprioception and was performed in the absence of additional auditory stimulation, tendon vibrations (80 Hz) were applied to postural and peripheral sites (paraspinal muscles, Achilles tendon, and forearm) while visual observation was maintained under the VMT conditions (Figure 8). Participants were again asked to report the occurrence of visual VPS. This procedure, involving different proprioceptive stimulation sites, aimed to determine whether visual losses were specifically associated with stimulation of muscles involved in postural control, with VPS occurrence during forearm muscle vibration interpreted as reflecting a more global involvement of proprioceptive processing. VPS were analyzed in terms of percentage of occurrence, mean size, and spatial distribution.

**Figure 9:**
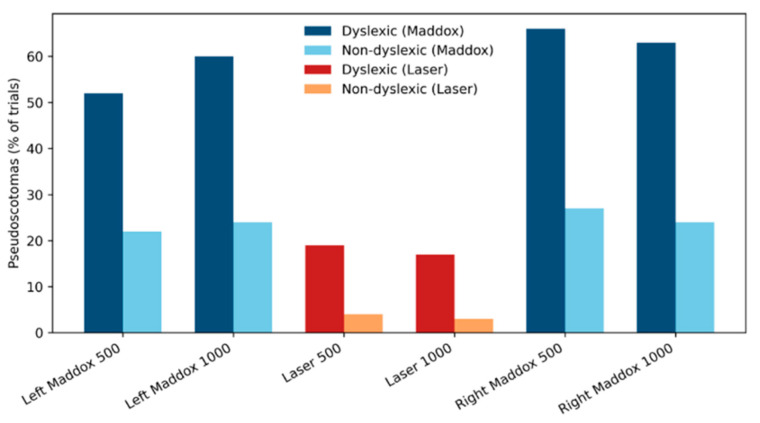
Frequency of pseudo-visual scotomas in typical readers and children with dyslexia during auditory stimulation, under modified binocular viewing conditions (left and right Maddox tests), or with intact binocular vision (laser).

No VPS were observed in the absence of auditory or proprioceptive sensory stimulation. In the audiovisual task, auditory stimulation under the Maddox condition induced VPS that were significantly more frequent and larger in children with dyslexia than in control participants (*p* < 0.001). These effects were independent of sound frequency and of the eye in front of which the Maddox screen was placed. VPS size followed a modulation pattern similar to that observed for their frequency of occurrence, and no consistent spatial organization of VPS was identified (Figure 9).

**Figure 10:**
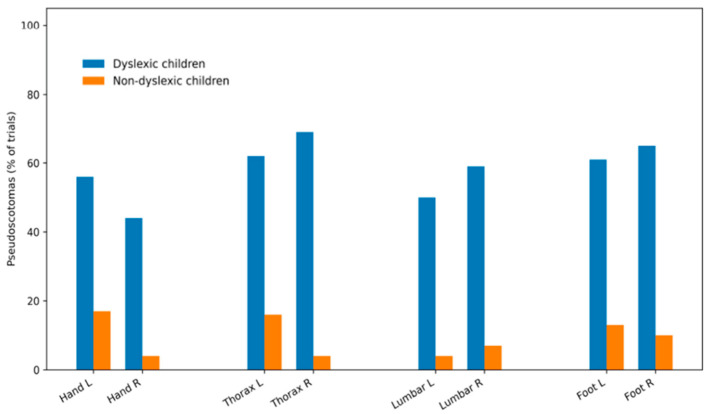
Pseudo-scotoma frequency during the vertical Maddox test in dyslexic and non-dyslexic children under proprioceptive stimulation of postural or non-postural muscles.

In the visuo-proprioceptive task, children with dyslexia exhibited a mean VPS occurrence rate of 60.3 ± 9.1%, compared with 15.9 ± 7.0% in control children (*p* < 0.001). VPS size was significantly greater in the dyslexic group, with no effect of vibration site (Figure 10).

VPS characteristics were not correlated with ophthalmological parameters (magnitude of vertical heterophorias, convergence ability) or with dyslexia severity. In contrast, VPS measures were significantly associated with the Maddox lability index, suggesting that VPS magnitude may constitute a reliable marker of global proprioceptive integrity or vulnerability.

To our knowledge, this study provides one of the first experimental observations suggesting that transient visual losses can be induced by auditory or proprioceptive stimulation at a distance from the ocular system. Although this phenomenon is not specific to dyslexia, it is markedly amplified in children with dyslexia. Visual losses emerge when oculomotor balance is artificially disrupted, and ocular proprioception plays an increased regulatory role in maintaining retinal image fusion.

Although VPS occurrence was not directly correlated with reading performance, these findings highlight the central role that both ocular and general proprioception may play in multisensory integration disturbances in children with dyslexia. Such transient visual losses are likely to interfere with the establishment and automatization of phoneme–grapheme associations, which are fundamental to reading acquisition.

## 4. Extending the Framework: Sleep–Wake Regulation

The following section extends the discussion toward nocturnal physiological regulation. The observations presented here remain exploratory and are intended to generate hypotheses regarding possible interactions between sleep-related processes and daytime sensorimotor stability. In this perspective, sensorimotor regulation may extend beyond its daytime manifestations to influence physiological processes occurring during sleep, highlighting the potential importance of the sleep–wake cycle in learning-related functions.

### 4.1. Respiratory Dynamics: Diaphragmatic Impact of Proprioceptive Dysregulation

Beyond its primary ventilatory function, the diaphragm acts as a central hub within the network of breathing, contributing to postural control, spinal stabilization, and sensorimotor integration through coordinated interactions with axial and respiratory muscles [59]. The diaphragm, as the primary respiratory muscle, plays a role in trunk postural control through its anatomical attachments to the lumbar spine and rib cage. Its mechanical efficiency depends on a constant balance between respiratory drive from bulbopontine centers and postural control networks [60]. Any disruption of this balance affects both breathing and postural stability.

**Figure 11:**
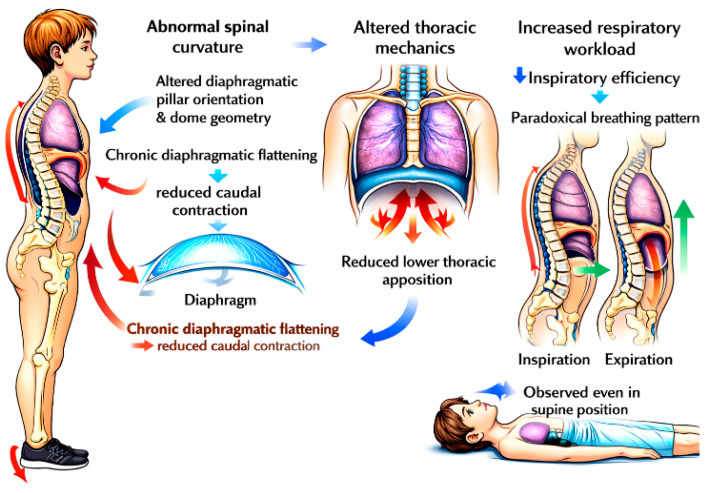
Respiratory Effects of Distorted Postural Vertical Perception in Dyslexic Children. Children load their heels and shift their center of pressure backward, despite perceiving themselves as standing upright. The body compensates with increased muscle tone, scoliotic posture, and forward shifts in both the pelvis and the head, which can make posture appear normal while maintaining chronic muscular tension. The diaphragm is continuously recruited as a postural stabilizer, restricting normal abdominal breathing; this dysfunction persists even when lying down, including during sleep, leading to inefficient thoracic respiration and sustained sympathetic activation.

In children with dyslexia, abnormal spinal curvatures alter diaphragmatic pillar orientation and dome geometry, frequently resulting in chronic diaphragmatic flattening. This reduces lower thoracic apposition and redirects contractile forces inward rather than caudally, thereby increasing respiratory workload and reducing inspiratory efficiency. As a consequence, a paradoxical breathing pattern commonly develops, characterized by posterior abdominal wall displacement and/or thoracic depression during inspiration, with reversed movements during expiration [61]. This pattern is consistently observed clinically, including in the supine position (Figure 11).

However, it is important to emphasize that these observations remain primarily clinical and were not assessed using objective respiratory measurements, such as spirometry, respiratory inductance plethysmography, or electromyography. Consequently, they should be considered hypothesis-generating observations requiring formal physiological validation.

### 4.2. The Orofacial-Postural Interface: Mouth Breathing and Craniofacial Morphology

Postural adaptations in children with dyslexia are frequently associated with a forward head posture, which favors persistent mouth breathing and an immature swallowing pattern characterized by low tongue positioning [62]. These functional disturbances interfere with craniofacial growth and are commonly associated with dentoskeletal dysmorphoses, particularly Class II malocclusions [63]. These features are relatively common in the general pediatric population and are not specific to dyslexia.

The relationship between craniofacial morphology and oral function is reciprocal [64]. Dysmorphoses that reduce upper airway patency, such as palatal constriction or retrognathia, further promote forward head posture as a compensatory strategy to maintain ventilation [65]. In parallel, chronic mouth breathing may contribute to adenotonsillar hypertrophy, exacerbating both respiratory and swallowing dysfunctions [66].

Furthermore, several studies have demonstrated significant associations between altered oral sensory processing—particularly lingual tactile perception and oral stereognosis—and the presence of malocclusions, especially in individuals exhibiting atypical orofacial habits [67,68,69,70]. Given the high prevalence of orofacial dysmorphoses in children with dyslexia, these abnormalities should be evaluated within a sensorimotor framework rather than through a purely structural or orthodontic perspective [71].

In the context of the present framework, these characteristics are therefore not interpreted as defining features of dyslexia but rather as potentially interacting sensorimotor and respiratory factors that may contribute to broader regulatory differences in some individuals.

### 4.3. Sleep Architecture and Its Role in Cognitive Consolidation

In children, sleep is organized into alternating cycles of slow-wave sleep (SWS) and rapid eye movement (REM) sleep [72]. SWS is essential for metabolic clearance and attentional regulation. In contrast, REM sleep supports neurogenesis in early development, neuroplasticity across the lifespan, and the progressive automatization of cognitive, perceptual, and motor skills [73,74,75,76,77]. In typically developing children, sleep is therefore critical for memory consolidation and lexical learning, processes that appear less efficient in children with dyslexia [78]. REM sleep integrity, therefore, plays a key role in shaping both the functional and anatomical organization of neurons [79]. Notably, children with dyslexia often exhibit atypical neural architectures [80]. Even at rest, their brains operate differently from those of typically developing individuals [81].

Physiologically, REM sleep is characterized by generalized muscle atonia affecting thoracic respiratory muscles, rendering the diaphragm the primary driver of ventilation [82]. Under these conditions, impaired diaphragmatic function cannot be adequately compensated by intercostal muscle recruitment, leading to sleep fragmentation with frequent brief arousals.

### 4.4. Sleep Pathophysiology in the Dyslexic Population

Children with developmental dyslexia are known to suffer from various sleep disorders, including difficulty falling asleep and staying asleep [83,84]. Clinically, persistent morning fatigue is frequently reported by dyslexic children and their families, regardless of attentional comorbidities or overall cognitive load. These observations suggest that sleep disorders may be a fundamental characteristic of dyslexia, potentially contributing to cognitive and attentional difficulties during the day.

This observation prompted an investigation of sleep characteristics in this population. Sleep was assessed in 109 children with dyslexia and 134 typically developing readers aged 8–10 years using the Sleep Disturbance Scale for Children (SDSC), a validated 33-item parent-report questionnaire [85]. Additional sleep-related signs identified during open-ended interviews with approximately fifty parents—such as morning headaches and atypical head position during sleep—were incorporated into the assessment.

In parallel, supplementary questions addressing muscular, spatial, and cognitive dimensions, initially described by Martins da Cunha as being associated with proprioceptive dysfunction, were administered to the children.

Item selection was refined through correlation analyses, internal consistency assessment (Cronbach’s alpha), and principal component analyses with Varimax rotation. The final validated version comprised 34 items, including 19 child-completed items and 15 parent-completed items (Table 1).

Statistical analyses focusing on sleep-related variables revealed significant between-group differences (*p* < 0.05) for 15 items related to sleep onset and sleep quality, with 7 items reaching a higher level of significance (*p* < 0.01). These results indicate a markedly altered sleep profile in children with dyslexia, consistent with previous reports of reduced REM sleep duration, prolonged sleep onset latency, and sleep architecture alterations correlated with dyslexia severity [84,85]. Given the crucial role of sleep in brain plasticity and attentional capacities, these findings highlight the importance of systematically assessing sleep—at least through a parental questionnaire—in children with dyslexia. However, the subjective nature of these responses underscores the need for a multimodal assessment that combines both subjective and objective measures, with polysomnography remaining the gold standard for sleep evaluation.

Statistical analysis of muscular, spatial, and perceptual signs identified 19 features that statistically distinguished the two groups of children.

Taken together, these findings led to the development of a questionnaire designed to subjectively assess the risk of dyslexia based on clinical signs observed during both daytime and nighttime. Each item is rated on a 1-to-5 scale according to the frequency of the reported signs, allowing the computation of an overall score. A global score ranging from 34 to 170 was derived and analyzed using receiver operating characteristic (ROC) curves, yielding three probability thresholds: >59 (high probability of dyslexia), >79 (very high probability), and <45 (very low probability). Scores above 79 increased the likelihood of dyslexia by a factor of 21 compared with those of typically developing readers. Despite certain methodological limitations, this questionnaire represents a simple and clinically relevant tool for diagnostic orientation. However, this questionnaire should be considered a preliminary screening instrument and requires validation in independent cohorts before any diagnostic use.

Finally, a complementary questionnaire revealed that over 30% of parents of children with dyslexia were treated with nocturnal positive airway pressure for obstructive sleep apnea, despite a relatively young mean age (43 years). This prevalence contrasts sharply with national data indicating a treatment rate of 2.3% in the general adult population in France (Santé Publique France, 2022), suggesting a potential familial or shared vulnerability affecting sleep-related respiratory control [86].

## 5. Toward an Embodied and Sensorimotor Perspective on Dyslexia

### 5.1. Interpretation of the Present Findings

Taken together, the results examined here do not support the existence of a single causal mechanism linking proprioception to developmental dyslexia. Rather, they suggest that variations in sensorimotor regulation may represent one of several interacting dimensions contributing to the heterogeneity of dyslexic profiles.

It is important to note that, according to current international diagnostic classifications (DSM-5-TR and ICD-11), major sensorimotor deficits remain exclusion criteria for the diagnosis of developmental dyslexia. Consequently, the present approach does not aim to redefine dyslexia as a primary sensorimotor disorder. Instead, it explores the possibility that subtle variations in sensorimotor regulation—although insufficient to constitute a classical neurological disorder—may interact with cognitive processes involved in reading acquisition.

From this perspective, sensorimotor regulation may influence the conditions under which perceptual, attentional, and linguistic processes operate during learning, thereby contributing to the variability observed among individuals with dyslexia.

### 5.2. Position Within Existing Theoretical Models

During the past decades, developmental dyslexia has primarily been studied within neuropsychological frameworks inspired by cognitive models of information processing [87]. In these approaches, cognition is typically described as the manipulation of abstract symbolic representations, and reading disorders are generally attributed to a specific deficit in phonological processing. Within this framework, dyslexia has been characterized as a persistent difficulty in establishing and automatizing grapheme–phoneme correspondences.

Subsequent developments in neuroscience have supported the emergence of connectionist models, in which cognitive and linguistic representations are viewed as distributed patterns of activation within large-scale neural networks. From this perspective, phonological difficulties in dyslexia are often interpreted as reflecting atypical connectivity within neural circuits supporting language and reading processes. Neuroimaging studies have indeed reported structural and functional differences in both gray and white matter in individuals with dyslexia [88].

However, the considerable interindividual variability observed across behavioral, cognitive, and neurobiological data argues against a single explanatory neural mechanism and is increasingly recognized as a defining feature of dyslexic phenotypes [89].

Within both cognitive and connectionist traditions, sensory and motor differences frequently reported in dyslexia—such as auditory processing differences, visual perceptual difficulties, or alterations in postural and motor coordination—have generally been considered associated features or comorbidities [90,91]. At the same time, increasing evidence from neuroimaging studies suggests that developmental dyslexia involves alterations in multiple brain networks, supporting the view that it may reflect broader neurodevelopmental differences rather than a single isolated deficit [92].

Early hypotheses emphasizing the potential contribution of perceptual-motor factors, including proposals highlighting alterations in proprioceptive and visual processing, have therefore remained relatively peripheral within dominant theoretical frameworks.

### 5.3. Embodied Cognition as an Integrative Framework

In contrast, embodied cognition frameworks propose that cognitive processes—including perception, language, and memory—are grounded in sensorimotor systems and emerge from their continuous interaction with the environment [5]. Within this perspective, abstract cognitive operations are shaped, though not determined, by sensorimotor experience accumulated throughout development.

Meaning attribution is therefore understood as relational and context-dependent, often described in terms of affordances. Bodily states, emotional regulation, and environmental constraints may modulate attentional and executive processes that support learning.

From this viewpoint, proprioception—given its role in motor control, spatial organization, and multisensory integration—may represent a relevant but still underexplored dimension in the study of developmental dyslexia.

Adopting such a perspective does not imply that dyslexia should be reduced to a sensorimotor disorder. Rather, it suggests that reading difficulties may emerge from the interaction of multiple cognitive, perceptual, and motor factors whose relative contributions likely vary across individuals.

### 5.4. Sleep–Wake Regulation and Nycthemeral Dynamics of Sensorimotor Processes

An additional dimension emerging from our studies concerns the frequent presence of sleep-related disturbances in a subset of dyslexic children. Clinical observations in our cohorts suggested that these disturbances were often associated with alterations in sensorimotor regulation, particularly involving postural control and diaphragmatic function.

Because breathing mechanics, upper airway stability, and axial postural tone are closely interconnected through proprioceptive pathways and brainstem regulatory circuits, subtle disruptions in these systems may influence nocturnal respiratory patterns and overall sleep quality [59].

From this perspective, the sleep–wake cycle may represent a nycthemeral dimension of sensorimotor regulation. Taken together, these observations suggest that sensorimotor regulation in developmental dyslexia may need to be examined across the entire 24-h cycle.

While most experimental studies focus exclusively on daytime cognitive or perceptual performance, the stabilization and consolidation of these processes largely depend on physiological mechanisms operating during sleep.

From this perspective, dyslexia may benefit from being studied not only as a cognitive learning disorder but also as a condition potentially involving regulatory dynamics that extend across both waking and sleeping states [75,76,77,78].

Consequently, even moderate disturbances of nocturnal regulation may influence learning efficiency and the stabilization of reading-related processes.

A growing body of research has also highlighted strong associations between sleep disturbances and attentional regulation, particularly in children with attention-deficit/hyperactivity disorder [93]. Because attentional fluctuations are frequently reported in dyslexia and may contribute to variability in reading performance, sleep-related regulatory processes may represent one pathway through which sensorimotor and physiological factors interact with attentional control mechanisms.

These observations should not be interpreted as indicating that sleep disturbances are specific to dyslexia. Rather, they suggest that alterations in sensorimotor and respiratory regulation during sleep may represent one component of a broader multifactorial framework contributing to developmental variability in reading acquisition.

Within such a perspective, the study of sleep–wake dynamics may therefore provide a useful complement to daytime investigations of sensorimotor functioning when attempting to understand the complex interactions underlying learning processes.

## 6. Limitations and Future Directions

Several limitations of the present clinical framework should be acknowledged. First, although this review integrates converging clinical observations and experimental findings accumulated over an extended period, much of the available evidence remains observational or correlational in nature. Consequently, the present framework does not establish a causal relationship between proprioceptive dysfunction and developmental reading disorders. Rather, it aims to contextualize existing findings within an embodied cognition perspective, emphasizing the potential role of sensorimotor regulation, multisensory integration, and sleep-related physiological mechanisms.

Second, developmental dyslexia is a heterogeneous condition, and the framework proposed here is not intended to apply uniformly to all individuals with reading disorders. Instead, it may be particularly relevant to subgroups presenting with marked sensorimotor, postural, or regulatory instabilities. This heterogeneity underscores the importance of refined phenotypic characterization integrating cognitive, sensorimotor, and physiological dimensions.

In addition, several clinical assessment tools discussed in this review—such as postural evaluations, proprioceptive stress tests, and sleep-related questionnaires—are informative at the clinical level but remain insufficiently standardized across research contexts. Although their relevance is supported by converging observations, further validation using objective neurophysiological and polysomnographic measures is required, particularly to better characterize the potential contribution of sleep-related disturbances.

Future research should therefore prioritize longitudinal and controlled studies combining standardized reading assessments with objective measurements of proprioceptive function, multisensory integration, and sleep architecture. Such integrative approaches may help clarify the temporal dynamics linking sensorimotor regulation, sleep-dependent plasticity, and learning processes across development.

Importantly, the present framework is not proposed as an alternative to established phonological or cognitive models of dyslexia. Rather, it should be viewed as a complementary perspective that may help guide future experimental and clinical investigations.

## 7. Conclusions: Toward an Embodied 24-h Perspective on Dyslexia

An increasing body of empirical and clinical observations suggests that proprioceptive processing and sensorimotor integration may be atypical in developmental dyslexia. Difficulties in visuospatial localization, the occurrence of functional visual pseudoscotomas, and alterations in postural and diaphragmatic regulation all point to subtle instabilities in multisensory processing. These instabilities may interact with mechanisms involved in reading acquisition, attentional control, and sleep regulation, and are broadly consistent with longstanding reports of sensory, motor, and attentional differences described in the dyslexia literature.

From this perspective, proprioceptive function should not be regarded as a competing explanation for dyslexia but rather as a complementary dimension that may help bridge existing theoretical frameworks, including cerebellar, magnocellular, visuospatial, and attentional models. Positioned at the interface between sensory input, motor control, and cognitive functioning, proprioception represents a relevant level of analysis for understanding how bodily regulation may influence learning conditions across development.

Importantly, proprioceptive function can be explored and potentially modulated through multidisciplinary clinical approaches that extend beyond reading-centered interventions. Preliminary observations suggest that targeted sensorimotor strategies may be associated with improvements in postural stability, attentional regulation, and reading-related performance. Although these findings remain exploratory, they highlight the potential relevance of considering bodily regulation within a broader learning context.

Overall, this synthesis supports a view of dyslexia compatible with contemporary embodied cognition frameworks, in which cognitive development emerges from dynamic interactions among sensory, motor, and perceptual systems. In this perspective, reading acquisition may depend not only on cognitive processes but also on the stability of sensorimotor regulation across the sleep–wake cycle. Considering dyslexia within this 24-h regulatory framework may help bridge daytime sensorimotor observations with sleep-dependent processes known to support learning and neuroplasticity. If alterations in proprioceptive regulation contribute to destabilizing sensorimotor integration across this cycle, they may represent a meaningful target for clinical recalibration and open new avenues for integrative and embodied approaches to reading remediation.

## Figures and Tables

**Table 1:** Questionnaire Assessing Daytime and Nighttime Indicators Distinguishing Dyslexic from Typical Readers.

For Each Symptom, Indicate the Number Corresponding to the Frequency 1 = Never, 2 = Occasionally (1 or 2 Times/Month), 3 = Sometimes (1 to 2 Times/Week), 4 = Often (3 to 5 Times a Week), 5 = Every Day.	
**15 Questions for PARENTS (after examining the child’s sleep for at least 3 h)**	**SCORE**
The child jerks or moves body parts as they fall asleep.	
The child has agitated daydream scenes when falling asleep.	
The child moves his legs a lot while sleeping, often changing position during the night, or kicking the bed covers.	
You have observed that your child sleepwalks.	
Your child has nightmares (terrors) that he can’t remember the next morning.	
He has great difficulty waking up in the morning.	
The child feels unable to move around and feels very tired when waking up in the morning.	
The child is sleepy during the day (easily falls asleep in the car, quiet, …).	
The child salivates a lot at night, or there are drool traces on the pillow in the morning.	
The child complains of a headache in the morning.	
The child breathes with its mouth open while sleeping.	
The child still pees or often gets up at night to go to the toilet.	
The child has trouble remembering lessons learned the night before (even though he knew them the night before).	
The child tends to be a little sleepy at times at school.	
The child has an abnormal head position while sleeping (head tilted back and in extension).	
**19 Questions to be answered by the CHILD**	
**Muscular dimension**	
Do you feel tired even if you haven’t exerted yourself physically or mentally?	
Is it hard for you to stand by and do nothing?	
Do you get a headache after school?	
Do you have recurring lower or upper back pain?	
Do your legs ever hurt?	
Is it hard for you to stare at a text (or a person) up close?	
Do you ever see double when you’re tired, after reading a text?	
Do you get out of breath quickly when you exert yourself (for example, as soon as you run)?	
Can you see blurred up close after reading a few lines (with your glasses, if you have them)?	
**Spatial dimension**	
Is it difficult for you to walk on something narrow (a beam, for example)?	
Is it difficult for you to catch an object on the first try—a ball, for example?	
Do you fall easily or twist your ankles?	
You bite your tongue or cheeks when you eat.	
Do you bump into simple obstacles (doorframes, for example, …) as if you didn’t perceive the space around you properly?	
**Perceptive dimension**	
Do you feel like you’re reading without understanding what you’re reading?	
Do you find it hard to concentrate for long?	
When someone speaks to you, do you feel like you don’t really understand what you’re hearing?	
When you read, you have the impression that you can’t see properly: you skip words, you miss line breaks, …	
Is it hard for you to express an idea when you’re talking, and you have trouble constructing your sentences properly?	
**TOTAL SCORE—Enter the SUM of the scores for all 34 questions**	

## Data Availability

No new data were created or analyzed in this study.
